# Multilevel analysis of school anti-smoking education and current cigarette use among South African students

**DOI:** 10.11604/pamj.2017.26.37.7880

**Published:** 2017-01-24

**Authors:** Brandon Talley, Katherine Masyn, Rachna Chandora, Alana Vivolo-Kantor

**Affiliations:** 1 Georgia State University, School of Public Health, Atlanta, Georgia, USA

**Keywords:** Adolescent Health, smoking and tobacco use, tobacco control and policy, school health instruction, school based health promotion

## Abstract

**Introduction:**

South Africa (SA) implemented the Global Youth Tobacco Survey (GYTS) four times between 1999 and 2011. Data from the four surveys indicated that downward trends in cigarette use among students may have stalled. Understanding the effect of school anti-smoking education on current smoking among students within schools and variability across schools may provide important insights into policies aimed at preventing or reducing tobacco use among students. The objective was to assess the student- and school-level effects of students' exposure to school anti-smoking education on current cigarette use among the study population using the most recent wave of GYTS data in SA (2011).

**Methods:**

An analytic sample of students 13-15 years of age was selected (n=3,068) from the SA GYTS 2011. A taxonomy of two-level logistic regression models was fit to assess the relationship of various tobacco use, control, and exposure predictor variables on current cigarette smoking among the study population.

**Results:**

At the student-level in the full model, secondhand smoke (SHS) exposure, peer smoking, and ownership of a promotional item were significantly associated with higher risk of current smoking. At the school-level in the full model, average exposure to peer smoking was associated with significant increases in the prevalence of current cigarette use, while average family anti-smoking education was significantly associated with decreases in the outcome variable. School anti-smoking education was not a statistically significant predictor at the student- or school-levels.

**Conclusion:**

in this study, exposure to school anti-smoking education had no association with current cigarette smoking among the study population. Consistent with previous studies, having peers that smoked was highly associated with a student being a current smoker. Interestingly, at the school-level in the multilevel analysis, schools with higher rates of average family anti-smoking education had lower prevalence of current smoking. This finding has potential implications for tobacco control in SA, particularly if the school-level, family-centered protective effect can be operationalized as a prevention tool in the country's tobacco control program.

## Introduction

Smokers in countries represented by the World Health Organization's Regional Office for Africa (WHO AFRO) consumed 3% of the world's cigarettes annually, the lowest level of consumption by region in the world [1]. Despite comparatively low consumption, Méndez, Alshanqeety, and Warner predict smoking prevalence in the region could increase by nearly 40 percent between 2010 and 2030 if no additional tobacco policies are implemented [2]. In addition to the threat of rising consumption, countries in sub-Saharan Africa lag behind the other regions in implementation of tobacco control policies recommended by WHO [3]. In 2016, Member States represented by WHO AFRO had an overall implementation rate of WHO Framework Convention on Tobacco Control (WHO FCTC) compliant policies of 43 percent compared to 53 percent in the remaining WHO Regions [3].

While tobacco use across sub-Saharan Africa remains relatively low, South Africa (SA) had 5.5 million smokers in 2012-the highest number of smokers of any country in the Region [4]. Even though the reported number of smokers in the country is high, SA achieved notable wins in fighting the tobacco epidemic over the last several decades. In the overall population, cigarette consumption halved between 1991 and 2011 [5]. Among students in grades 8-10, cigarette smoking declined from 1999 to 2008 [6]. Unfortunately, these hard-won successes may be transitory. Declines in cigarette use stalled nationally among students between 2008 and 2011 [6]. Perhaps more telling, black students- which comprise the largest racial subpopulation of youth in SA-had cigarette smoking rates that remained unchanged from 1999 to 2008 and increased (albeit insignificantly) between 2008 and 2011 [6]. Such stalls may signal growth in tobacco consumption not only in SA but also across sub-Saharan Africa [2].

Notwithstanding the grim forecast, effective implementation of evidence-based tobacco control policies could prevent tobacco use among South African youth. Studies demonstrated school anti-smoking education potentially prevents students' initiation of tobacco use in both developed and developing countries [7–9]. In SA, however, findings from a randomized trial of two different school-based smoking prevention programs found no difference in 30-day smoking rates from baseline to 2-year follow-up among the study's high-school student population [10]. The lack of efficacy demonstrated in the SA trial raises questions about the effectiveness of school anti-smoking education programs implemented in the country.

To support further evaluation of programs implemented in the country, the current study assessed student- and school-level effects of school anti-smoking education on current cigarette smoking. The study population included students aged 13-15 years old from the most recent wave of SA's Global Youth Tobacco Survey (GYTS) conducted in 2011. In addition to school anti-smoking education, variables with known associations to current cigarette smoking among youth were examined. The inclusion of these variables provided an opportunity to evaluate the magnitude of important effects such as tobacco advertising and exposure to tobacco countermarketing on current cigarette smoking among students.

## Methods

**Study overview:** The GYTS, a component of the Global Tobacco Surveillance System (GTSS), is a school-based, cross-sectional survey that enhances countries' capacity to design, implement, and evaluate tobacco control interventions [11]. It also enables countries to report on compliance to articles of the WHO FCTC and implementation of the WHO MPOWER technical package [11]. The MPOWER technical package includes measures to Monitor tobacco use and prevention policies; Protect people from tobacco smoke; Offer help to quit tobacco use; Warn about the dangers of tobacco; Enforce bans on tobacco advertising, promotion, and sponsorship; and Raise tobacco taxes. In consultation with stakeholders, WHO and the US Centers for Disease Control and Prevention (CDC) developed a standard GYTS methodology for constructing the sampling frame; selecting schools and classes; preparing questionnaires; following consistent field procedures; and using consistent data management procedures for data processing and analysis [12]. The GYTS core questionnaire of 56 questions covers seven domains related to youth tobacco use: 1) knowledge and attitudes toward cigarette smoking; 2) prevalence of cigarette smoking and other tobacco use; 3) role of media and advertising in cigarette use; 4) access to cigarettes; 5) tobacco-related school curriculum; 6) environmental tobacco smoke; and 7) cessation. Countries have an opportunity to add optional questions [12].

**Procedure:** Each country implementing GYTS is required to follow a standardized protocol for sampling and recruitment. The GYTS protocol uses a two-stage cluster sample design to produce nationally representative data of students aged 13-15 years. In the first stage, schools across a country are selected with a probability proportional to enrollment size. The schools selected are then recruited to participate in the survey. In the second stage, classes are randomly selected from each school. All students in selected classes are eligible and invited to participate in the anonymous, self-reported survey. In the current study, 171 schools participated in the survey. The questionnaire was administered during one class period and took approximately 30-40 minutes to complete. Administration procedures were followed to protect students´ privacy and anonymity. Students were reminded that their participation was voluntary, and they could stop completing the questionnaire at any time during administration. The overall response rate was 69.1% for all students surveyed in the SA 2011 GYTS.

**Study participants:** Data for the current study are from the full GYTS conducted in SA during 2011; the full study included a total of 10,833 students in grades 8-11. For the current study, students aged 13-15 years were included as outlined in the GYTS protocol (n=3947). In addition to the age range restriction, the study examined only cases with complete information on covariates of interests, which resulted in an analytic sample of n=3068. To evaluate the potential impact of missing data, we compared pairwise present associations between each predictor and the outcome for all students aged 13-15 years (n=3947) with the final analytic sample (n=3068). We found negligible differences in the magnitude of effects between the two samples. In the analytic sample, the mean age of study participants was 14.40 (SD = 0.70). The majority of study participants were black (71.0%) and female (57.9%). The current smoking rate among the study population was 11.6% (SD =0. 32).

**Measures:** The study included three groups of variables: outcome, controls, and predictors. The outcome of interest was current cigarette smoking. Controls included sex, age, and race. Predictor variables were included a priori based on previous research. Measures were self-reported, and each is more fully described in Table 1 and below.

**Table 1:** Study measures and variables created from the 2011 South Africa Global Youth Tobacco Survey (GYTS) items

Study measure		GYTS survey item	GYTS item responses	Dichotomous study variable
Current cigarette smoking status		During the past 30 days (one month), on how many days did you smoke cigarettes?	0 days 1 to 2 days 3 to 5 days 6 to 9 days 10 to 19 days 20 to 29 days All 30 days	No=0 days Yes>0 days
Exposure to secondhand smoke	Outside the home	During the past 7 days, on how many days have people smoked in your home, in your presence?	0 days 1 to 2 days 3 to 4 days 5 to 6 days 7 days	No=0 days Yes>0 days
	Inside the home	During the past 7 days, on how many days have people smoked in your presence, in places other than in your home?		
Parental or peer smoking	Peer smoking	Do your parents/guardians smoke?	Both my parents/guardians do not smoke Both my parents/guardians smoke Only my father/male guardian smokes Only my mother/female guardian smokes I don’t know	No= ‘Both my parents/guardians do not smoke’ or ‘I don’t know’ Yes=any other response
	Parental/guardian smoking	Do any of your closest friends smoke cigarettes?	None of them Some of them Most of them All of them	No=’None of them’ Yes=any other response
Knowledge of smoking harms		Do you think cigarette smoking is harmful to your health? Do you think the smoke from other people's cigarettes is harmful to you?	Definitely not Probably not Probably yes Definitely yes	No=’definitely not’ for both items Yes=any other response for either item
Ownership of cigarette branded promotional item		Do you have something (tshirt, pen, backpack, cap etc.) with a cigarette brand logo on it?	No Yes	No Yes
Offered a free cigarette by a cigarette company representative		Has a cigarette representative (someone working for a cigarette company) ever offered you a free cigarette?	No Yes	No Yes
Exposure to countermarketing		During the past 30 days (one month), how many antismoking media messages (e.g. television, radio, billboards, posters, newspapers, magazines, movies) have you seen or heard?	None A few A lot	No=none/never for both items Yes=any other response for either item
		When you go to sports events, fairs, concerts, community events, or social gatherings, how often do you see antismoking messages?	I never go to…or Never A lot Sometimes	
School anti-smoking education		During this school year, were you taught in any of your classes about the dangers of smoking? During this school year, did you discuss in any of your classes the reasons why people your age smoke? During this school year, were you taught in any of your classes about the effects of smoking (such as it makes your teeth yellow, causes wrinkles, or makes you smell bad)?	No Not sure Yes	No=’No’ or ‘Not sure’ for all three items Yes=’Yes’ for any of the three items
Family anti-smoking education		Has anyone in your family discussed the harmful effects of smoking with you?	No Yes	No Yes

Note: study measures listed do not include control variables (province, race, sex, and age)

**Current cigarette smoking:** Current cigarette smoking was assessed by asking students how many days they smoked in the past 30 days. Response options were ordinal. Previous research on GYTS at the global level recommended dichotomizing the ordinal responses by recoding any response of 1 day or greater as “yes” and 0 days to “no” for current smoking [13]. We empirically examined the appropriateness of the binary recoding prior to conducting the multilevel analysis by comparing the estimated effects of each covariate on dichotomous response options (binary logistic regression) to estimated effects on the original ordinal scale (ordinal logistic regression). The empirical findings indicated no substantive difference in effects; as a result, the binary coding for current smoking was used throughout analyses in the current study for clarity of presentation.

**Control variables:** Students reported sex, age, and race. Sex was assessed by asking students to report sex as “Male” or “Female”. Students had eight response options for age: “11 years or younger, 12, 13, 14, 15, 16, 17, or 18 years or older”. For the current study, only respondents aged 13-15 years were included. Age was treated as a continuous variable for all analyses. To measure self-identified race, students were asked: “During Apartheid, people were placed into different race groups. In which race group do you think that you would have been placed?”. Response options for the race item included “Black/African, Coloured, Indian, White, Other, and Don't Know”. Dummy variables for race were constructed for use in all analyses; “Black/African”race was the reference group for the dummy variable.

**Observing cigarette smoking:** Observing cigarette use included four variables: two secondhand smoke (SHS) exposure variables (inside/outside the home) and the parent/peer smoking variables. In the global analysis of GYTS conducted by Koh and colleagues, the two items for SHS exposure were collapsed into one binary SHS exposure variable. Similarly, the two parents/guardian and peers smoking items were collapsed into a single binary parent/peer smokers variable [13]. Prior research demonstrated that observing others smoke-particularly peers- is a predictor of initiation of cigarette smoking among adolescents [14, 15]. As a result, rather than assume a homogeneous, non-cumulative effect for the SHS exposure and parent/peer smokers variables, we examined these four items for differential effects prior to conducting the multilevel analyses. All four items had a significant and distinct association with current smoking and, thus, each was included in the multilevel analyses according to type of exposure: SHS inside/outside the home and observing parent/peer smoking.

**Knowledge of the harms of smoking:** The knowledge of the harms of smoking construct was assessed with two items measured on an ordinal scale. One item focused on the student's own smoking. The other focused on the student's exposure to SHS. These items were collapsed into a single binary variable and recoded as “yes” if respondents answered “yes” to either of the individual items.

**Exposure to tobacco advertising, promotions, and sponsorship:** The construct for exposure to tobacco advertising, promotion, or sponsorship included two items measured on a binary scale: owning a cigarette branded promotional item and offered a free cigarette by a cigarette company representative.

**Exposure to countermarketing:** The countermarketing construct was assessed with two items that measured frequency of exposure to anti-smoking messages on different nominal scales. The variables were collapsed into one binary variable. A response of any level of exposure to either item was considered a “yes” response in the binary recoding.

**Exposure to anti-smoking education:** The exposure to anti-smoking education construct was comprised of two sets of study measures: school anti-smoking education and family anti-smoking education. Three items asked about school-based education on the dangers of, reasons for, and effects of smoking cigarettes. Response options included “no,” “yes,” and “not sure.” The set of school anti-smoking education items were empirically evaluated to determine the appropriateness of combining the “not sure” responses with either the “yes” or “no” responses. Preliminary analysis indicated no significant difference in effect between the “no” and “not sure” responses for each item; thus, the two response options were collapsed for each item. Subsequent to this evaluation and using methods described by Koh et al., the three items were then dichotomized into a single school anti-smoking education variable if the respondent answered “yes” to any of the three items [13]. The family anti-smoking education study measure consisted of a single binary item that assessed whether or not family members discussed the harmful effects of smoking.

**School-level variables:** F or the multilevel analysis, several school-level variables were derived. School-level means were derived for age, sex, race and the predictor variables. Age, which was a continuous variable with a range of 13-15 years, was computed as a mean for each school. Sex, race, and the predictor variables had binary response options; consequently, the school-level variable aggregates represented the school-level proportions of each endorsement category, e.g., proportion of males. In addition to the derived variables, eight non-derived dummy variable indicators were created to represent the nine provinces in SA.

**Data analysis:** Data management was performed using SAS 9.4. Descriptive statistics were computed and model building was conducted using Mplus 7.0 with the two-level analysis type. Prior to analysis and as previously mentioned, most of the GYTS survey items included in the current study were dichotomized to reflect methods from previous research Table 1. For all analyses, a weighting factor was applied to each student record to adjust for the probability of selection, non-response, and post-stratification adjustment to population estimates. Sample statistics were computed for the outcome variable; age, race, and sex at the student- and school-levels; and predictors at the student- and school-levels.

A taxonomy of two-level logistic multilevel models was then fit. The multilevel models accounted for the nested nature of the data. Age, sex, and race were controlled at the student- and school-levels for each model. Similarly, province was controlled at the school-level for all models specified. Nine models in total were specified. The first set of eight models examined the unique effects of individual study measures: 1) SHS exposure; 2) parent/peer smoking; 3) knowledge of the harms of smoking; 4) ownership of cigarette brand promotional item; 5) offered a free cigarette by a cigarette company representative; 6) exposure to countermarketing; 7) school anti-smoking education; and 8) family anti-smoking education. The full model examined the adjusted effect of all study measures simultaneously. Lastly, an intraclass correlation coefficient (ICC) was calculated.

## Results

Table 2 displays descriptive statistics. The ICC was .20, which is high and indicated 20 percent of the total variance in smoking is explained at the school-level. Table 3 displays unstandardized coefficients and R^2^ values for multilevel models 1-8 and the full model. In addition to the R^2^ values presented for models 1-8 and the full model, two baseline R^2^ values were estimated with models that included only the controls (age, sex, and race) at the student-level and the controls plus province at the school-level. At the student-level controlling for age, sex, and race, variables from models 1-6 were significantly associated with increases in current smoking (p < 0.05). Peer smoking, one of the variables included in model 2, had the greatest effect. Notably, the predictor of interest for this study- school anti-smoking education-was not associated with current smoking. When assessing the full model with controls at the student-level, peer smoking remained the variable most strongly associated with current smoking (B: 1.630, p < 0.05). However, the variables for parent/guardian smoking, offered a free cigarette by a cigarette company representative, and exposure to countermarketing were no longer statistically significant. For models 1-8, comparisons of student-level R2 values at baseline with each model indicated that model 2 (parent/peer smoking) uniquely explained the highest amount of variance (21 percent) in the outcome at this level. The full model explained approximately 32 percent of variance at the student-level, which corresponded to approximately 26 of the total variance. At the school-level in models 1-8 controlling for age, sex, race, and province, only the unique effects of average peer smoking (model 2) and average family anti-smoking education (model 8) were significantly associated with current smoking (B: 1.544, -1.854; p < 0.05). Although statistically significant, the school-level peer smoking effect was expected given the strong association of peer smoking with current smoking at the student-level. The average family anti-smoking education had an unexpectedly strong protective effect. The full model with controls indicated the magnitude of average peer smoking and average family anti-smoking education was nearly equal but in opposite directions. Similar to the student-level model, having a peer that smoked uniquely explained the most variance in the outcome at the school-level in models 1-8. The full model explained 89 percent of variance at the school-level, which corresponded to approximately 5 percent of the total variance. Province, which was only included at the school-level, uniquely explained approximately 5 percent of the total variance.

**Table 2:** Descriptive statistics on study variables for participants aged 13-15 years

	Percent or mean (SD)	
**Variables**	Student-Level (n=3068)	School-Level^a^ (n=171)
Outcome		
Current smoking	11.6	11.0 (1.2)
Demographics		
Age	14.4 (.70)	14.5 (.38)
Sex		
Male	42.1	37.4 (2.1)
Female	57.9	59.9 (1.5)
Race		
Black	71.0	73.7 (3.3)
Coloured	12.5	11.7 (.8)
Indian	1.2	1.2 (.4)
White	9.6	7.9 (2.1)
Other	.5	.4 (.3)
Don't Know	5.2	5.2 (1.0)
Exposure to secondhand smoke		
Inside the home	30.7	31.0 (2.1)
Outside the home	40.3	40.7 (2.2)
Parent/peer smoking		
Parent/guardian smoking	33.2	33.3 (2.2)
Peer smoking	30.7	31.4 (2.3)
Knowledge of the harms of smoking	85.2	84.8 (2.0)
Ownership of cigarette brand promotional item	12.4	11.1 (1.0)
Offered a free cigarette by a cigarette company representative	10.9	11.7 (1.3)
Exposure to countermarketing	82.4	84.0 (1.5)
School anti-smoking education	72.4	73.4 (2.0)
Family anti-smoking education	51.9	52.9 (2.3)

a School-level variables defined as school means

**Table 3:** Unstandardized coefficients (B) and R^2^ values for multilevel analysis of study variables and the full model (n=3068)

	Model								
Study Measure	1	2	3	4	5	6	7	8	Full
Student-Level									
Exposure to secondhand smoke									
Inside the home	0.824+++								0.633+++
Outside the home	0.937+++								0.741+++
Parent/peer smoking									
Parent/guardian smoking		0.450++							0.120
Peer smoking		1.818++							1.630+++
Knowledge of the harms of smoking			0.981+++						0.603+
Ownership of cigarette brand promotional item				0.591++					0.421+
Offered a free cigarette by a cigarette company representative					0.498+				0.333
Exposure to countermarketing						0.703+++			0.367
School anti-smoking education							0.249		0.087
Family anti-smoking education								0.202	0.087
R^2^ (Baseline with age, sex, and race only: 0.04++)	0.17+++	0.21+++	0.08++	0.05++	0.05++	0.06++	0.05++	0.05++	0.32+++
School-Level^a^									
Exposure to secondhand smoke									
Inside the home	0.532								0.134
Outside the home	0.853								1.131
Parent/peer smoking									
Parent/guardian smoking		0.009							-0.413
Peer smoking		1.544++							1.525+
Knowledge of the harms of smoking			-0.429						-1.031
Ownership of cigarette brand promotional item				0.187					-0.222
Offered a free cigarette by a cigarette company representative					0.149				-1.218
Exposure to countermarketing						0.343			-0.578
School anti-smoking education							-0.569		-0.003
Family anti-smoking education								-1.854++	-1.660+
R^2^ (Baseline with age, sex, race, and province only: 0.66+++)^b^	0.66+++	0.91+++	0.65+++	0.66+++	0.65+++	0.64+++	0.66+++	0.76+++	0.89+++

Note: all estimates adjusted for province, race, sex, and age,

+ *P <* 0.05,

++ *P <* 0.01,

+++ *P <* 0.001

a School-level variables defined as school means

Table 4 presents standardized and unstandardized coefficients as well as odds ratios for the full model. Unstandardized coefficients (previously detailed above with results from Table 3) provided the basis for computing odds ratios at the student-level. Students exposed to SHS inside the home had nearly two times the odds of being a current smoker. Outside the home, students exposed to SHS had more than a two-fold increase in odds of current smoking. Surprisingly, students indicating they had knowledge of the harms of smoking had over 1.5 times the odds of current smoking. Similar findings were seen for those who owned a cigarette branded promotional item. Particularly striking, students with peers that smoked had a five-fold increase in odds of current smoking.

**Table 4:** Results for full multilevel model (n=3068)

Study Measure	B	SE	p-value	OR (95% CI)	β
Student-level					
Exposure to secondhand smoke					
**Inside the home**	**0.633**	**0.181**	***<* 0.001**	**1.884 (1.322, 2.685)**	**0.133**
**Outside the home**	**0.741**	**0.155**	***<* 0.001**	**2.098 (1.550, 2.840)**	**0.166**
Parental or peer smoking					
Parental smoking	0.120	0.175	0.493	1.128 (0.800, 1.589)	0.026
**Peer smoking**	**1.630**	**0.148**	***<* 0.001**	**5.102 (3.818, 6.819)**	**0.342**
**Knowledge of smoking harms**	**0.603**	**0.296**	**0.042**	**1.828 (1.023, 3.267)**	**0.098**
**Ownership of cigarette branded promotional item**	**0.421**	**0.180**	**0.019**	**1.523 (1.070, 2.168)**	**0.063**
Offered a free cigarette by a cigarette company representative	0.333	0.185	0.073	1.395 (0.970, 2.006)	0.047
Exposure to countermarketing	0.367	0.256	0.152	1.444 (0.874, 2.386)	0.064
School anti-smoking education	0.087	0.145	0.549	1.091 (0.820, 1.451)	0.018
Family anti-smoking education	0.087	0.137	0.524	1.091 (0.834, 1.428)	0.020
School-Level^a^					
Exposure to secondhand smoke					
Inside the home	0.134	0.773	0.862		0.036
Outside the home	1.131	0.672	0.092		0.315
Parent/peer smoking					
Parent/guardian smoking	-0.413	0.814	0.612		-0.115
**Peer smoking**	**1.525**	**0.610**	**0.012**		**0.454**
Knowledge of the harms of smoking	-1.031	0.911	0.258		-0.257
Ownership of cigarette brand promotional item	-0.222	1.049	0.832		-0.028
Offered a free cigarette by a cigarette company representative	-1.218	1.051	0.247		-0.193
Exposure to countermarketing	-0.578	0.847	0.495		-0.114
School anti-smoking education	-0.003	0.547	0.996		-0.001
**Family anti-smoking education**	**-1.660**	**0.672**	**0.014**		**-0.482**

Note: all estimates adjusted for province, race, sex, and age

a School-level variables defined as school means

## Discussion

Overall, cigarette consumption has declined in SA over the last three decades; however, declines in student smoking slowed in recent years [6]. If these slowing declines mark a new era of increasing tobacco use among South Africans, the country will face greater rates of tobacco-related morbidity and mortality in the future. Although reasons for the slowing declines in students are unclear, our findings suggest that school anti-smoking education did little to prevent or reduce smoking among students in SA in 2011 at the student- or school-levels. The current study's results on school anti-smoking education were similar to other research conducted in Africa [16].

In countries across sub-Saharan Africa, research demonstrated peer smoking is a significant predictor of current smoking among students [16–18]. Not surprisingly, we found students that had peers who smoked had much greater odds of being a current smoker. While the peer smoking results were expected, the protective effective of average family anti-smoking education on current smoking at the school-level has not been well documented with GYTS data. This protective effect alludes to a potential family-centered social dynamic that may prevent initiation of cigarette smoking in youth within certain schools.

Previous research demonstrated that such social dynamics have been associated with lower rates of smoking [19–21]. For example, in a social network analysis of the longitudinal Framingham Heart Study, researchers identified smoking-cessation cascades where entire connected clusters of study participants quit smoking in near unison [21]. The Framingham findings suggested decisions and intent to quit smoking were facilitated by network phenomena, i.e., the choice or intent to quit reflected not only individual behavior change but also evolving normative beliefs linked to attitude changes toward smoking within interconnected groups.

While the smoking-cessation cascades and social cohesion research focused primarily on tobacco cessation, it is useful to consider the influence of social or network phenomena on preventing initiation of tobacco use among youth. In the current study, a social phenomenon- the protective effect of school-level average family anti-smoking education-may hint at a latent family-centered, school-specific social dynamic that curbs initiation of cigarette smoking among individuals and their peers. Because adolescent populations have demonstrated difficulty quitting smoking even when tobacco was used infrequently [22, 23], leveraging the type of protective social phenomenon hinted at in the current study could reduce the high number of smokers in SA. Given the limited research on such social phenomena in the tobacco control context, the influence of the family-school social network on preventing tobacco use among youth at the school-level warrants further study.

**Limitations:** The current study is subject to several limitations. Because the survey included youth who attended school and were present on the day of survey administration, it may not be representative of all youth aged 13-15 years. As a cross-sectional survey, effects identified in the study are suggestive rather than causal. The self-reported data may introduce bias due to over- or under-reporting in response to survey items. Lastly, the items in GYTS were not a direct measure of school anti-smoking education; as a result, issues such as fidelity of delivery of school anti-smoking education could not be quantified.

## Conclusion

In this study, exposure to school anti-smoking education had no association with current cigarette smoking among the study population. Consistent with previous studies, having peers that smoked was highly associated with a student being a current smoker. Interestingly, at the school-level in the multilevel analysis, schools with higher rates of average family anti-smoking education had lower prevalence of current smoking. This finding has potential implications for tobacco control in SA, particularly if the school-level, family-centered protective effect can be operationalized as a prevention tool in the country's tobacco control program.

### What is known about this topic

- Globally, the effectiveness of school anti-smoking education is mixed depending on a variety of factors. In South Africa, recent clinical trials showed no efficacy in school-based smoking prevention programs.

### What this study adds

- Consistent with previous research, school anti-smoking education had no association with current cigarette smoking among the study population; however, schools with higher rates of average family anti-smoking education had lower prevalence of current smoking;
- This unexpected finding could suggest a school-level, family-centered protective effect that can potentially be operationalized as a prevention tool in the country's tobacco control program.

## References

[1] Eriksen M, Mackay J, Ross H (2012). The Tobacco Atlas..

[2] Méndez D, Alshanqeety Omar, Warner Kenneth (2013). The potential impact of smoking control policies on future global smoking trends. Tobacco Control..

[3] Husain MJ, English LM, Ramanandraibe N (2016). An overview of tobacco control and prevention policy status in Africa. Preventive Medicine..

[4] Ng M, Freeman MK, Fleming TD, Robinson M, Dwyer-Lindgren L, Thomson B (2014). Smoking Prevalence and Cigarette Consumption in 187 Countries. 1980-2012 JAMA. Journal of the American Medical Association..

[5] Jha P, Peto R (2014). Global Effects of Smoking, of Quitting, and of Taxing Tobacco. New England Journal of Medicine..

[6] Reddy PJ, Sewpaul R, Yach D, Resnicow K, Sifunda S, Mthembu Z, Mbewu A (2013). A decade of tobacco control: The South African case of politics, health policy, health promotion and behaviour change. South African Medical Journal..

[7] Hollingworth WC, Cohen David, Hawkins James, Hughes Rachael, Moore Laurence, Holliday Jo (2012). Reducing Smoking in Adolescents: Cost-Effectiveness Results From the Cluster Randomized ASSIST (A Stop Smoking In Schools Trial). Nicotine & Tobacco Research..

[8] Thomas RE, McLellan J, Perera R (2013). School-based programmes for preventing smoking: Evidence-Based Child Health. A Cochrane Review Journal..

[9] Pierce JP, White V, Emery S (2012). What public health strategies are needed to reduce smoking initiation?. Tobacco Control..

[10] Resnicow KR, Sasiragha P, James S, Omardien RG, Kambaran NS, Langner HG (2008). Comparison of Two School-Based Smoking Prevention Programs among South African High School Students: Results of a Randomized Trial. Annals of Behavioral Medicine..

[11] US Centers for Disease Control and Prevention About GTSS..

[12] World Health Organization Global Youth Tobacco Survey..

[13] Koh HK, Alpert HR, Judge CM, Caughey RW, Elqura LJ, Connolly GN (2011). Understanding worldwide youth attitudes towards smoke-free policies: an analysis of the Global Youth Tobacco Survey. Tobacco Control..

[14] Huang GC, Unger JB, Soto D, Fujimoto K, Pentz MA, Jordan-Marsh M (2014). Peer influences: the impact of online and offline friendship networks on adolescent smoking and alcohol use. Journal of Adolescent Health..

[15] Kelly AB, O'Flaherty M, Connor JP, Homel R, Toumbourou JW, Patton GC (2011). The influence of parents, siblings and peers on pre- and early-teen smoking: A multilevel model. Drug and Alcohol Review..

[16] Mamudu HM, Veeranki SP, John RM (2013). Tobacco Use Among School-Going Adolescents (11-17 Years) in Ghana. Nicotine & Tobacco Research..

[17] Veeranki SP, Mamudu HM, John RM, Ouma AEO (2015). Prevalence and correlates of tobacco use among school-going adolescents in Madagascar. Journal of Epidemiology and Global Health..

[18] Agaku IT, Alpert HR, Vardavas CI, Adisa AO, Connolly GN (2014). Use of smokeless tobacco and cigarettes among Nigerian youths: implications for tobacco control policies in Africa. Journal of Substance Use..

[19] Fleischer NL, Lozano P, Arillo Santillán E, Reynales Shigematsu LM, Thrasher JF (2015). The impact of neighbourhood violence and social cohesion on smoking behaviours among a cohort of smokers in Mexico. Journal of Epidemiology & Community Health..

[20] Patterson JM, Eberly LE, Ding Y, Hargreaves M (2004). Associations of Smoking Prevalence with Individual and Area Level Social Cohesion. Journal of Epidemiology Community Health..

[21] Christakis NA, Fowler JH (2008). The collective dynamics of smoking in a large social network. New England Journal of Medicine..

[22] Soteriades ESS, George, Talias, Michael A, Warren, Charles W, DiFranza, Joseph R (2011). Children's loss of autonomy over smoking: the global youth tobacco survey. Tobacco Control..

[23] Ursprung WWSAD, DiFranza JR (2010). The loss of autonomy over smoking in relation to lifetime cigarette consumption. Addictive Behaviors..

